# Developing capacity in identifying cost-effective interventions to prevent and reduce obesity in China

**DOI:** 10.1080/16549716.2025.2463794

**Published:** 2025-03-10

**Authors:** Angela M. Jackson-Morris, Suying Chang, Christina L. Meyer, Guansheng Ma

**Affiliations:** a Center for Global Noncommunicable Diseases, RTI International, Durham, NC, USA; b Child Health and Development Section, The United Nations Fund for Children (UNICEF), Beijing, China; c Center for Applied Economics and Strategy, RTI International, Durham, NC, USA; d Health Science Center, Peking University, Beijing, China

**Keywords:** Capacity building, health planning, noncommunicable diseases, child obesity, adolescent obesity, health economics, policy making, global health, investment case, middle-income countries

## Abstract

Obesity is associated with multiple noncommunicable diseases and has increased rapidly worldwide. Population obesity in China grew fourfold between 1993 and 2015, increasing most rapidly among children and adolescents. Cost-effective policies and programs delivered over time and at scale are required to change this trajectory, yet application of methodologies to identify such interventions have been sparse. UNICEF China and Peking University together identified the need to strengthen the intervention evidence available to policymakers and to build stakeholders’ knowledge and skills. Investment cases combine a review of intervention evidence, policy landscape assessment, and economic modelling to identify cost-effective interventions suited to a specific context. A training and mentorship program aimed to build awareness, knowledge, and skills about this methodology to encourage its use to support decision making and planning to address obesity. Program participants reported increased knowledge of analytical methods to identify contextually relevant cost-effective obesity interventions (92% of evaluation respondents), and 82% reported increased knowledge of evidence-based obesity interventions. 79% reported confidence to apply the learning in their job roles. Training and mentorship can enhance stakeholder knowledge, skills, and confidence to apply investment case methodology to develop economic evidence to strengthen the basis of obesity policy and program commissioning.

## Paper Context

- **Main findings**: Capacity building can strengthen knowledge, skills, and confidence to develop economic evidence to strengthen the basis of obesity policy and program commissioning.
- **Added knowledge**: Capacity building in economic evidence development was welcomed by diverse stakeholders to address an existing gap and inform policy and program decisions for obesity prevention.
- **Global health impact for policy and action**: Building countries’ capacity to review and assess the cost-effectiveness of diverse intervention types can contribute to increasing the commissioning of evidence-based, effective, policy and program interventions.

## Background

Overweight and obesity has become a major global public health issue in recent decades, linked to multiple noncommunicable diseases (NCDs), and affects populations in countries where previously undernutrition or micronutrient deficiency was the exclusive focus. Rapidly increasing obesity among children and adolescents is particular concerning, given the potential risk of associated NCDs to affect a sizeable proportion of future generations. Until relatively recently, NCD programs and capacities have been under-developed and underfunded in many low- and middle-income countries (LMICs) compared to other health programs, such as for infectious diseases [1,2]. One of the LMICs facing a rapid growth in obesity and overweight prevalence is China. From 2000 to 2020 obesity and overweight prevalence among children and adolescents aged 0–19 in China surged by 400% to an estimated 37.9% in 2020, surpassing other Western Pacific and upper-middle-income countries [3].

To address this surge, China has implemented physical activity and nutrition education programs in schools and issued national guidance to pregnant mothers and schools. A significant limitation has been the absence of interventions to address obesogenic environments, as well as interventions in primary care. This dominant intervention model originated prior to the escalation in obesity and lifestyle changes in recent decades [4]. Despite highly developed clinical research on NCDs from Chinese researchers and institutions, robust research on effective health promotion and prevention has been more limited [4,5], with obesity prevention research one of the least developed areas [6]. Contextual information from the literature and stakeholder discussions indicated that government agency policymakers, commissioners, managers, and academics were largely unfamiliar with, or did not consider certain obesity intervention types [7], and applying analytical methodologies to assess potential obesity strategies in China was rare.

In response, UNICEF commissioned a study to strengthen the available evidence regarding cost-effective obesity intervention priorities. The research to identify a set of context-appropriate, cost-effective, national-level interventions was undertaken by RTI International in partnership with Peking University (PKU), with input from a panel of Chinese experts [7,8]. It used China-specific and global evidence on effective interventions and was informed by an analysis of the policy landscape, that highlighted limited prior use of economic analysis to identify cost-effective obesity prevention intervention priorities.

Following the study’s completion, UNICEF China and PKU agreed that building stakeholder capacity to undertake this type of analysis could provide tailored robust evidence to policymakers. This was important given policymakers’ general preference for locally tailored evidence on NCDs [9]. Such capacities could enable future updates of the national-level analysis and recommendations as the intervention evidence grows and contextual data changes. A capacity building program of training and mentorship was commissioned with RTI International to develop awareness, knowledge, and skills about economic modelling to identify contextually relevant cost-effective obesity prevention interventions. In the context of a gradual increase in the number of LMIC NCD departments, strategies, academic research centers, and LMIC-focused research funding calls for NCD capacity building [10], this article shares a capacity building approach and recommendations for developing local health economic research capacity to contribute to evidence-based obesity policy and program commissioning in settings where obesity is becoming a major public health issue.

## Approach

The program’s short-term aim was to build knowledge and skills about investment cases as an applied analytical methodology, and to share examples from the national study to illustrate the diverse interventions that had been assessed as cost-effective in the Chinese context.

**Figure 1.**
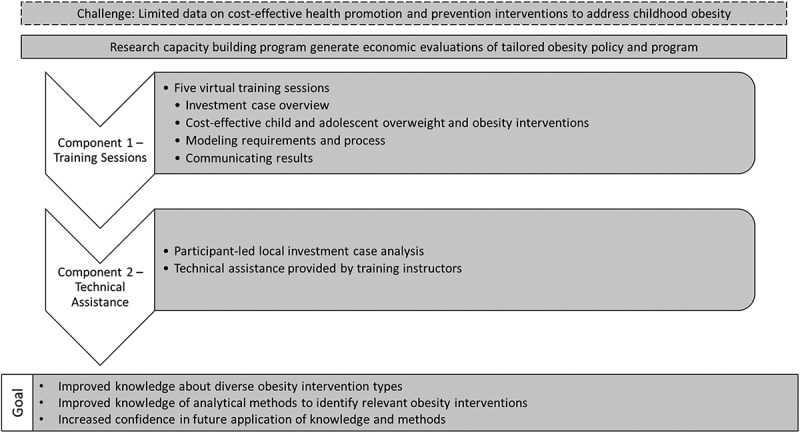
Capacity building program to aimed to build awareness, knowledge, and skills about this methodology to encourage its use to support decision making and planning to address obesity.

The capacity building program was designed with two components (Figure 1). Component one was a series of five virtual training sessions included: an investment case overview; evidence on cost-effective child and adolescent overweight and obesity interventions; modelling requirements and process; and communicating results. This was delivered to the full participant cohort (*n* = 32), including personnel from various regions and with a range of job roles considered to have potential to apply the program’s content: i) government nutrition institute staff who advise on policy and, or commission or manage programs and research (*n* = 9); ii) academics with obesity prevention interest (e.g. epidemiologists, physicians) or economists (*n* = 10); and iii) students (*n* = 13). PKU researchers recruited participants from national government institutes (*n* = 2) and universities (*n* = 6). A second component was technical assistance to support a small team of Beijing-based obesity prevention researchers who participated in the training to ‘learn by doing’ (*n* = 3) by applying the training to develop a local investment case and generate an output to catalyze discussions with stakeholders.

## Findings

An online post-program evaluation survey was developed for program participants to complete anonymously. The questionnaire was purposely limited to three questions to assess critical outcomes while encouraging a high response rate (a 100% response rate was achieved), including a quantitative rating on a 5-point Likert scale and free-text space to qualitatively explain the rating. Respondents indicated the degree they believed the capacity building had enhanced their knowledge and skills about the analytical methodology and its uses, and their confidence and potential scope to apply these. A high proportion of respondents rated the training as very high or high impact (Likert 4 or 5) in increasing their knowledge about diverse obesity intervention types (92%) and the analytical methods to identify these relevant to national or local contexts (82%) (Table 1). There was no marked difference in the results between different job backgrounds or those who only received the universal session and those who also undertook the practical learning component. 79% of all respondents reported confidence that they would be able to apply some of the knowledge in their current or future work. This proportion was substantial albeit lower than for knowledge acquisition, as might be expected given the various job roles and capacity for direct application. The high ratings overall may reflect that participants were selected for both the relevance of their roles or disciplines and their known prior interest or involvement to at least some degree in the topic. Illustrative quotations from respondents reflect these findings:

I will do related research from the perspectives I learned. (Participant 1)

**Table 1.** Capacity building evaluation survey scores (*n* = 32).

	Survey Responses (*n* = 32)		
*Question (5-point Likert scale: 1=No impact; 5=Significant impact)*	*Workshop participant average score*	*Likert scale scores 4–5* *(% of respondents)*	*Likert scale scores 1–2* *(% of respondents)*
On a scale from 1–5, to what extent did the capacity building and/or investment case add to your knowledge of different types of obesity interventions?	4.4	92%	0%
On a scale from 1–5, to what extent did the capacity building and/or investment case strengthen your knowledge of analytical methods to identify cost-effective approaches for obesity interventions?	4.2	82%	3%
On a scale from 1–5, to what extent do you believe you can apply the theories and/or skills that you gained from the capacity building and/or investment case in your current or future work?	4.2	79%	0%

The tool would be very useful for facilitating decision making on early prevention of obesity, but we still need more detailed guidance on how to use it. (Participant 2)

We can apply the theories and measurement skills to the current policies and effects on childhood obesity prevention, and develop better cost-effective policy in the future. (Participant 3)

The intermediate impact will be the extent to which the methods are applied in practice. Qualitative feedback suggested strengthening some aspects within the wider research ecosystem would also aid application in practice. These included bilingual skills or translation technology to enable inclusion of both Chinese and non-Chinese language research studies, and critical review skills to assist identification of robust studies for the economic analysis.

It’s paramount to highlight the importance of adeptness in bilingual literature reviews…enabling the identification and selection of research articles that align with envisioned intervention strategies. This proficiency is not merely about staying abreast of current research trends but also involves a nuanced understanding of how to distinguish the most pertinent and credible studies for our purposes. (Participant 4)

As per any policy, the longer-term goal to increase the number of cost-effective national or local obesity interventions implemented will be moderated by wider factors, including the political acceptability of certain intervention types [11]. The wider literature suggests that economic evidence can influence perceptions of, and inclinations towards specific policies [12]. For example, UNICEF engaged with policymakers using economic evidence on obesity interventions, and believe it had a major contributory influence legislation to improve school food environments [13,14]. Similar stakeholder engagement was undertaken using the China national investment case on child and adolescent obesity [7], generating 13 media reports and anecdotal feedback suggesting greater interest among officials in interventions for a healthy food environment [15]. Following the capacity building UNICEF and PKU indicated strong motivation to undertake further analyses with stakeholders in cities or provinces across China to identify context-relevant, cost-effective interventions to address child and adolescent obesity.

## Lessons learnt

Capacity building programs can augment stakeholder awareness, knowledge, and skills on important public health issues, such as obesity, where the evidence base is underdeveloped. Slow policy recognition and concurrently limited funding has stymied obesity capacity building programs in LMICs, resulting in skill and knowledge gaps in various sectors. Recommendations from obesity prevention programs and research that has been undertaken in LMICs highlight the substantial unmet need for capacity development to upskill policymakers and researchers [16,17]. The World Obesity Federation’s ‘SCOPE’ no-fee online training modules include policy considerations in their content and encourage policymaker participation [18]. Yet, while recommending the use of analytical approaches, including investment cases, the online training on the ‘how to’ is not included [18]. Our findings from the China program suggest that training and skills mentorship in analytical methodologies, such as investment cases, can develop knowledge and skills that create awareness about the limitations of existing evidence, and can contribute to future research, intervention design, and commissioning.

Funders should support similar capacity building initiatives across LMICs to build the analytical skills that can guide future obesity prevention and management interventions. LMIC institutions can also contribute by fostering inter-disciplinary collaboration between, for example, health economists, social scientists, and public health scientists – all of whose skills can play a role in reversing the current trajectory of obesity prevalence.

## References

[1] World Health Organization. Assessing national capacity for the prevention and control of noncommunicable diseases: report of the 2021 global survey [internet]. 2023 [cited 2024 Apr 8]. Available from: https://www.who.int/publications-detail-redirect/9789240071698

[2] Kassa M, Grace J, Kassa M, et al. The global burden and perspectives on non-communicable diseases (NCDs) and the prevention, data availability and systems approach of NCDs in low-resource countries. Public health in developing countries - challenges and opportunities [Internet]. IntechOpen; 2019 [cited 2024 Apr 8]. Available from: https://www.intechopen.com/chapters/69468

[3] Okunogbe A, Nugent R, Spencer G, et al. Economic impacts of overweight and obesity: current and future estimates for 161 countries. BMJ Glob Health [Internet]. 2022 [cited 2022 Oct 5];7:e009773. doi: 10.1136/bmjgh-2022-009773 PMC9494015 36130777

[4] Ma L, Wen X, Xue H, et al. National childhood obesity‐related intervention systems and intervention programs in China in 1949 to 2020: a narrative review. Obesity [Internet]. 2022 [cited 2022 Jun 8];30:320–6. doi: 10.1002/oby.23316 35088555

[5] Xue Y, Ruan Z, Ung COL, et al. Policy analysis of system responses to addressing and reversing the obesity trend in China: a documentary research. BMC Public Health [Internet]. 2023 [cited 2024 May 8];23:1198. doi: 10.1186/s12889-023-15890-7 37344819 PMC10283163

[6] Wang Y, Zhao L, Gao L, et al. Health policy and public health implications of obesity in China. Lancet Diabetes & Endocrinol [Internet]. 2021;9:446–461. doi: 10.1016/S2213-8587(21)00118-2 34097869

[7] Ma G, Meyer CL, Jackson-Morris A, et al. The return on investment for the prevention and treatment of childhood and adolescent overweight and obesity in China: a modelling study. Lancet Reg Health – West Pac [Internet]. 2024 [cited 2024 May 28];43:100977. doi: 10.1016/j.lanwpc.2023.100977 38456086 PMC10920044

[8] UNICEF China. The return on investment for the prevention and treatment of obesity [Internet]. 2023 [cited 2024 Apr 8]. Available from: https://www.unicef.cn/en/reports/return-investment-prevention-and-treatment-obesity-0

[9] Jackson-Morris AM, Miranda J, Nugent R. Tailoring off-the-shelf global evidence with local implementation research can boost action on overweight and obesity. Lancet Global Health [Internet]. 2023 [cited 2023 Nov 27];11:e826–e827. doi: 10.1016/S2214-109X(23)00173-0 37031686

[10] Global Alliance for Chronic Diseases. Global alliance for chronic diseases [Internet]. GACD; 2024 [cited 2024 May 8]. Available from: https://www.gacd.org/

[11] Gunarathne A, Spiller A, Risius A. Public acceptability of government interventions to reduce obesity: policy effectiveness, policy fairness, government trust and political ideology. Proc Nutr Soc [Internet]. 2020 [cited 2024 May 28];79:E128. doi: 10.1017/S0029665120000762

[12] Cullerton K, Donnet T, Lee A, et al. Effective advocacy strategies for influencing government nutrition policy: a conceptual model. Int J Behav Nutr Phys Act [Internet]. 2018 [cited 2024 May 28];15:83. doi: 10.1186/s12966-018-0716-y 30170610 PMC6119246

[13] Gloria E. México enfrenta otro gran problema, alerta la Unicef [Mexico faces another big problem, warns Unicef] [Internet]. Enfoque Noticias; 2023 [cited 2024 May 28]. Available from: https://enfoquenoticias.com.mx/mexico-enfrenta-otro-gran-problema-alerta-la-unicef/

[14] Pacheco AL. Senado avala iniciativa de Entornos escolares saludables en México [Senate endorses healthy school environments initiative in Mexico] [Internet]. NVI Noticias; 2023 [cited 2024 Jan 12]. Available from: https://nvinoticias.com/nacional/senado-avala-iniciativa-de-entornos-escolares-saludables-en-mexico/153702

[15] Chen M. Intervention urged amid spike in child obesity rate [Internet]. China Daily; 2024 [cited 2024 Oct 31]. Available from: https://www.chinadaily.com.cn/a/202401/17/WS65a70a73a3105f21a507ca34.html

[16] Reeve E, Bell C, Sacks G, et al. Lessons for strengthening policymaking for obesity and diet-related noncommunicable disease prevention: a narrative synthesis of policy literature from the Western Pacific region. Obes Rev [Internet]. 2024 [cited 2024 Oct 22];25:e13651. doi: 10.1111/obr.13651 37905309

[17] Parra DC, Vorkoper S, Kohl HW III, et al. Research capacity for childhood obesity prevention in Latin America: an area for growth. Obes Rev [Internet]. 2017 [cited 2023 Jan 6];18:39–46. doi: 10.1111/obr.12579 28741908

[18] World Obesity Federation. Strategic centre for obesity professional education (SCOPE) [Internet]. World Obesity Federation; 2024 [cited 2024 Oct 22]. Available from: https://www.worldobesity.org/training-and-events/scope

